# Management of appendiceal mass and abscess in children; early appendectomy or initial non-operative treatment? A systematic review and meta-analysis

**DOI:** 10.1007/s00464-020-07822-y

**Published:** 2020-07-24

**Authors:** Paul van Amstel, Tania C. Sluckin, Tim van Amstel, Johanna H. van der Lee, Ralph de Vries, Joep P. M. Derikx, Roel Bakx, L. W. Ernest van Heurn, Ramon R. Gorter

**Affiliations:** 1 Emma Children’s Hospital, Amsterdam UMC, University of Amsterdam & Vrije Universiteit Amsterdam, Department of Pediatric Surgery, Amsterdam, The Netherlands; 2 Emma Children’s Hospital, Amsterdam UMC, University of Amsterdam, Pediatric Clinical Research Office Amsterdam, Amsterdam, The Netherlands; 3 Knowledge Institute of the Dutch Association of Medical Specialists, Utrecht, The Netherlands; 4 Vrije Universiteit Amsterdam, University Library, Amsterdam, The Netherlands

**Keywords:** Appendicitis, Children, Appendiceal mass, Appendiceal abscess, Non-operative treatment, Appendectomy

## Abstract

**Background:**

Appendiceal mass and abscess and its treatment are associated with significant morbidity and high costs. Still, the optimal treatment strategy is the point of debate. Therefore, this systematic review and meta-analysis aimed to compare overall complications between initial non-operative treatment (NOT) and early appendectomy (EA) in children with appendiceal mass and/or abscess.

**Methods:**

Pubmed and Embase were searched. Only randomized controlled trials and prospective or historical cohort studies that compared NOT with EA in children with appendiceal mass or abscess in terms of complications were eligible for inclusion. Risk of bias was assessed. Primary outcome was the overall complication rate. Secondary, length of stay and readmission rate were investigated. A meta-analysis of overall complications associated with both treatment strategies was performed.

**Results:**

14 of 7083 screened studies were selected, including 1022 children in the NOT group and 333 in the EA group. Duration of follow-up ranged between four weeks and 12 years. Risk of bias was moderate in four and serious in 10 studies. NOT was associated with a lower overall complication rate (risk ratio (RR) 0.37 [95% confidence interval (CI) 0.21–0.65]). However, NOT led to increased length of stay (mean difference varied between 0.2 and 8.4 days) and higher readmission rate (RR 1.75 [95%CI 0.79–3.89]), although not significantly. Interval appendectomy after NOT was performed as a routine procedure in all but one study. This study found a recurrence rate of 34% in a group of 38 patients during a follow-up period of 3.4 ± 1.7 years.

**Conclusion:**

NOT may reduce the overall complication rate compared to EA, but the evidence is very uncertain. As evidence is scarce, and of low level, and heterogeneity between studies is substantial, the results should be interpreted with caution. Large prospective studies are needed to determine the optimal treatment strategy for children with appendiceal mass and/or abscess.

**Electronic supplementary material:**

The online version of this article (10.1007/s00464-020-07822-y) contains supplementary material, which is available to authorized users.

## Background

In the pediatric population, complex appendicitis is common, especially in children < 7 years old [1]. Approximately 35% of children with acute appendicitis present with the complex form. Although a uniform definition of complex appendicitis is lacking, in most studies it represents a spectrum ranging from gangrenous appendicitis to perforated appendicitis with generalized peritonitis. Generally, complex appendicitis can be divided into two main subgroups: without appendiceal mass and/or abscess (75%) and with appendiceal mass and/or abscess (25%) [1].

In general, complex appendicitis and its treatment are associated with significant morbidity (complications occurring in up to 30% of patients), prolonged length of hospital stay, and high costs [2]. Heterogeneity in the treatment of children with an appendiceal mass and/or abscess (e.g., initially non-operative treatment versus early appendectomy) still exists in daily practice. Some (pediatric) surgeons prefer initially non-operative treatment consisting of intravenous antibiotics (with or without percutaneous drainage), since this strategy is associated with less complications [3]. Others favor early appendectomy because a second trip to the hospital in order to perform an interval appendectomy can be avoided and, if the interval appendectomy is not performed as same day procedure, a shorter length of hospital stay is expected. This is one of the main reasons why early appendectomy is recommended by the Dutch guideline on the diagnosis and treatment of acute complex appendicitis as well [4]. This recommendation is merely based on expert opinion and in contrast to the limited and low-quality evidence.

In 2010 Simillis et al. published a meta-analysis that focused specifically on the treatment of appendiceal mass and abscess in both the adult and pediatric population. This meta-analysis could only include eight low-quality studies in the pediatric population and found that initial non-operative treatment was associated with a lower rate of overall complications (OR 0.21: 95%CI 0.11–0.38), wound infections (OR 0.22: 95%CI 0.07–0.66), and abscess formation (OR 0.11: 95%CI 0.04–0.35) compared to early appendectomy [3]. Since then, additional studies regarding the treatment of appendiceal mass and abscess in the pediatric population have been published that might provide novel insights [5, 6].

Therefore the aim of this systematic review and meta-analysis is to provide a complete overview of available literature regarding the treatment of the specific group of children presenting with appendiceal mass and abscess (identified according to predefined criteria) and to evaluate the effect of initial non-operative treatment (iv antibiotics with or without percutaneous drainage) (NOT) compared to early appendectomy (EA) on the rate of complications.

## Methods

The protocol of this systematic review and meta-analysis was registered at PROSPERO: International prospective register of systematic reviews with identification number CRD42018083522. This systematic review and meta-analysis was reported according to the Preferred Reporting Items for Systematic reviews and Meta-analysis (PRISMA) guidelines [7]. Ethical approval and written informed consent were not required, as this study only analyzed previously published data.

### Type of studies

All studies comparing EA with initial non-operative treatment for the management of appendiceal mass or abscess in children younger than 18 years were eligible for inclusion. Randomized controlled trials, prospective cohort studies and retrospective cohort studies were included in the review, whereas case series, case reports, letters to the editor, and conference abstracts were excluded. Language was restricted to English, German, French, and Dutch. Only studies that reported on our primary outcome, complication rate associated with both treatment strategies, were eligible for inclusion.

### Type of participants

Participants were children (< 18 years old) with complex appendicitis with the subtype of appendiceal mass and/or abscess. Only studies that defined their population at least with the terms ‘appendiceal mass/phlegmon’ or ‘appendiceal abscess’ were included. For further specification of these terms, definitions used in the original studies were followed.

### Types of interventions

Intervention: Initial NOT strategy consisting of administration of intravenous antibiotics (with or without percutaneous drainage) with in-hospital monitoring and administration of pain medication followed or not followed by interval appendectomy. Duration and type of antibiotics were not defined.

Comparison: Operative treatment strategy, consisting of an immediate (< 48 h after presentation) laparoscopic or open appendectomy with perioperative care according to local protocol. Studies comparing delayed appendectomy (> 48 h after presentation) as primary treatment strategy with NOT were excluded.

### Search methods

A comprehensive search was performed in the bibliographic databases PubMed and Embase.com in collaboration with our experienced medical librarian (RV). Databases were searched from inception up to November 7th 2019. The following terms were used (including synonyms and closely related words) as index terms or free text words: “Appendix”, “Appendectomy”, “Laparoscopy”, “Children”. A detailed search strategy is shown in Online Appendix 1. The reference lists of all included articles were cross-checked for identification of additionally relevant studies.

### Study selection and data extraction

Two reviewers (PA, TS) selected eligible articles independently; these were initially screened on title and abstract according to the predefined inclusion and exclusion criteria. Following this initial selection full texts were screened. After final selection of the included articles, two independent reviewers (PA, TS) extracted data using a predefined data extraction form. Data extraction included the following variables, but this list is not exhaustive: general information (author, year, methodology, patient characteristics, definition of appendiceal mass and/or abscess, treatment strategies, follow-up), primary outcome (complication rate), and secondary outcomes (i.e., length of hospital stay, recurrent appendicitis, and readmission rates). Discrepancies in both study selection and data extraction were resolved by consensus, and in case of disagreement a third reviewer was consulted (RG). Authors were contacted by email in case of missing outcome data. Correspondence did not lead to additional data.

### Outcomes and definitions

The primary outcome parameter was the overall complication rate in both treatment groups, defined as the proportion of patients experiencing at least one complication.

Complications of NOT included but were not limited to failure of NOT, i.e., patients not responding to the initial antibiotic treatment (with or without initial percutaneous drainage) and thus requiring additional interventions (e.g., additional drainage procedures, delayed appendectomy), and complications after interval appendectomy (as defined by the original authors).

Complications of operative treatment included but were not limited to extensive bowel resection and reoperations (as defined by the original authors).

Overall complications in both treatment groups included but were not limited to intra-abdominal abscess formation (IAA), superficial site infection (SSI) and ileus, as defined by the original authors.

If possible, complications were listed according to the Clavien–Dindo scale [8]. Grade 1: Any deviation from normal postoperative course without the need for farmacological treatment or a surgical/radiological intervention, Grade 2: Requiring farmacological treatment, Grade 3: Requiring surgical/radiological intervention, Grade 4: Life-threatening complication requiring ICU-admittance, Grade 5: Death of a patient.

Secondary outcomes included initial and total length of hospital stay (days) (total length of stay included interval appendectomy), readmission rate (defined as number of discharged patients that were admitted to the hospital again with complaints related to the previously experienced appendicitis), recurrent appendicitis (defined as number of patients who experienced symptomatic recurrence of disease with histopathologically proven recurrent appendicitis after completion of the initial course of antibiotics), number and type of imaging studies (ultrasound, Computed Tomography (CT), and Magnetic Resonance Imaging (MRI)), usage of pain medication (number of doses and type of pain medication (acetaminophen, non-steroidal anti-inflammatory drugs (NSAIDs), and morphine)), unexpected findings during surgery or at histopathological examination, number of surgical and/or radiological interventions, and Quality of Life (as defined by the original authors).

### Risk of bias and quality of evidence assessment

Two reviewers (PA, TA) applied the Cochrane Collaboration’s Risk of Bias Tool 2.0 for randomized controlled trials and the Risk Of Bias In Non-randomised Studies of Interventions (ROBINS-I) tool depending on the study design [9, 10]. Specifically bias due to confounding and bias in selection of participants were of importance. Bias due to confounding was considered low in randomized controlled trials, and moderate in prospective cohort studies and retrospective cohort studies that adjusted for baseline characteristics. Retrospective cohort studies that did not adjust for baseline characteristics were assessed as serious risk of bias. For all included studies risk of bias due to selection was assessed as low, moderate or serious according to the moment that patients were diagnosed with appendiceal mass or abscess:

- Low risk of selection bias: all patients included in the study were diagnosed with appendiceal mass or abscess (by physical examination or imaging) before the start of the intervention (i.e., non-operative treatment or early appendectomy).
- Moderate risk of selection bias: at least 50% of patients, but not all were diagnosed before the start of the intervention. Meaning that less than 50% of patients were diagnosed with appendiceal mass or abscess during or after the intervention.
- Serious risk of selection bias: less than 50% of patients had a diagnosis of appendiceal mass or abscess before the start of the intervention. Thus more than 50% of patients were diagnosed with appendiceal mass or abscess during or after the intervention. Both risk of bias tools were applied on the outcome level (overall complication rate) independently by the two authors. Conflicts were resolved by discussion until consensus. In case of persistent disagreement a third author (RG) was consulted.

Evidence of the studies and their original conclusions were rated according to the Oxford Centre for Evidence-Based Medicine Levels of Evidence Table [11].

Certainty of the evidence and conclusions of this systematic review and meta-analysis was evaluated using the Grading of Recommendations Assessment, Development and Evaluation (GRADE) method. GRADEpro software was used to create a Summary of Findings table for all pooled outcome measures and sensitivity analyses of studies at moderate risk of bias. The five GRADE considerations (study limitations, consistency of effect, imprecision, indirectness, and publication bias) were used to grade the evidence and conclusions. All decisions to downgrade the quality of the evidence were justified using footnotes.

### Data analysis

In case less than 50% of included studies reported on one of the outcome measures, meta-analyses were not performed. Furthermore, if statistical heterogeneity exceeded 70%, it was decided not to show the pooled effect estimate. Review Manager version 5.3.5 was used for the performance of the meta-analyses. The Mantel–Haenszel method was used to compute risk ratios and their corresponding 95% CI for dichotomous outcomes and to calculate weighted mean differences with 95% CI for continuous data. Means and variances were calculated according to the Cochrane Handbook for Systematic Reviews of Intervention [9]. Heterogeneity was assessed with the Higgins *I*^2^ inconsistency test. When *I*^2^ was more than 50%, statistical heterogeneity was considered substantial. Meta-analyses were performed using a random-effects model. Publication bias was assessed with funnel plots.

Sensitivity analyses were performed to examine the treatment effects on the primary outcome (overall complications) excluding studies with serious risk of bias (i.e., perioperative selection of patients), studies published before 2000, and studies without a well described definition of appendiceal abscess or mass. For the secondary outcomes sensitivity analyses were limited to studies with low or moderate risk of bias, by excluding the studies with serious risk of bias, and to studies published after 2000. Studies were divided into those reporting on patients presenting with appendiceal mass, those focusing on appendiceal abscess, and those analyzing a combination of both. Subsequently, overall complications were analyzed for these subgroups of studies.

Additionally an analysis was performed wherein the primary outcome (overall complication rate) was divided in IAA, wound infection, and ileus.

## Results

### Search

**Fig. 1:**
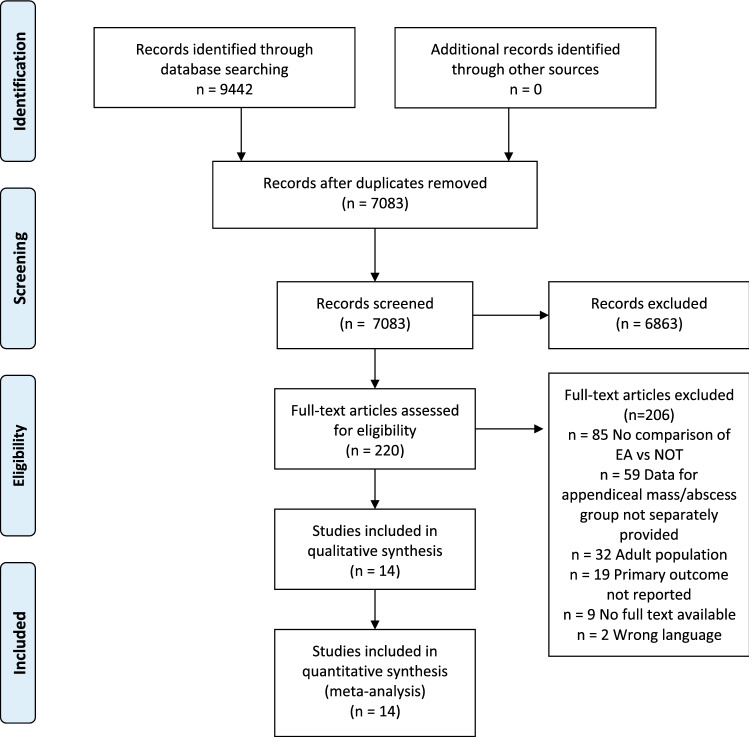
PRISMA flow diagram of the study selection

The search yielded 9442 articles, of which 4438 were found in Pubmed and 5004 in Embase. After removal of duplicates, 7083 articles were screened for title and abstract and 220 studies were assessed for full text. 206 articles were excluded because of various reasons. Fourteen studies were included in the systematic review and meta-analysis [5, 6, 12–23]. See Fig. 1 for a flowchart of the study selection.

### Included studies

The general characteristics of the included studies are shown in Table 1. Designs of the studies were one pilot randomized controlled trial [6], two non-randomized prospective studies [5, 18], and 11 retrospective studies [12, 13, 15–23]. These 14 studies included 1355 children, of which 333 were included in the EA group with a median [range] of 19 patients [7–60] per study and 1022 in the NOT group with a median [range] of 32 patients [6–411] per study. The papers were published between 1969 and 2016. Follow-up ended for most studies after interval appendectomy with a range of four [22]—23 weeks [21] after initial NOT [6,12–19.] Two studies reported long-term follow-up of 3–12 years [5, 23].

**Table 1:** Characteristics of included studies

Study design		Inclusion criteria				Treatment strategies		Population (n = 1355)	NOT (n = 1022)	EA (n = 333)	Mean age (SD)		Follow-up		Author recommended treatment strategy
		Age (years)	Abscess or mass	Definition (absces/mass)	Diagnosis based on	NOT	EA				NOT	EA	NOT	EA	
Calvert (2014) [16]	Retrospective cohort	< 18	Both	Inflammatory mass with or without non-drainable fluid collection < 3 cm	Imaging and/or perioperative findings	IV AB (10 days) + PICC line	Immediate appendectomy	106	64	42	–	–	6–8 weeks	–	NOT (± IA)
Emil (2007) [17]	Retrospective cohort	< 18	Both	Mass, not further defined Abscess; dominant ring enhanced	Perioperative findings	IV AB + drainage (if necessary)	Urgent appendectomy	76	32	44	7.4 (4.2)	8.4 (4.2)	6–12 weeks	–	NOT + IA
Erdogan (2005) [20]	Retrospective cohort	< 18	Mass	Mass, not further defined	Palpable mass and/or imaging and/or perioperative findings	IV AB (7 days)	Operation soon after primary evaluation	40	21	19	–	–	2–3 months	–	NOT + IA
Furuya (2015) [21]	Retrospective cohort	4–13	Abscess	Well-circumscribed abscess on US or CT	Imaging	IV AB without drainage	Emergency appendectomy	31	16	15	8.1 (2.5)	8.7 (3.2)	4–23 weeks	–	NOT + IA
Gahukamble (1993) [22]	Retrospective cohort	< 12	Mass	Mass, not further defined	Palpable mass and/or perioperative findings	IV AB	Immediate appendectomy	66	59	7	–	–	4 weeks	–	NOT + IA
Gastrin (1969) [23]	Retrospective cohort	< 15	Abscess	Abscess, not further defined	Palpable abscess and/or perioperative findings	Antibiotic therapy	Appendectomy after diagnosis	42	29	19	–	–	2–12 years	2–12 years	NOT + IA
Gillick (2001) [12]	Retrospective cohort	< 18	Mass	Mass, not further defined	Palpable mass and/or imaging	IV AB + bed rest	Immediate appendectomy	427	411	16	–	–	4–6 weeks	–	NOT + IA
Handa (1997) [13]	Retrospective cohort	< 15	Abscess	Abscess: Palpable mass which is filled with fluid demonstrated by US or CT	History, physical exam and imaging	IV AB + bed rest	Early appendectomy	14	6	8	8.0 (3.3)	6.9 (1.7)	3 months	–	NOT + IA
Puri (1981) [14]	Retrospective cohort	< 3	Both	Mass and/or abscess, not further defined	Palpable mass/abscess	IV AB + bed rest	Appendectomy	47	31	16	–	–	4 weeks	–	NOT + IA
Roach (2007) [15]	Retrospective cohort	< 18	Both	Mass: Inflammatory mass without clearly defined fluid-filled abscess Abscess: No definition	Imaging or perioperative findings or histopathological examination	IV AB + drainage	Immediate appendectomy + abscess drainage	92	32	60	–	–	6 weeks	–	NOT + IA
Samuel (2002) [18]	Prospective cohort	< 18	Both	Mass and/or abscess, not further defined	Palpable mass and/or imaging	IV AB	Appendectomy at presentation	82	48	34	6.8 (3.0)	6.9 (3.2)	Mean 10 (± 4) weeks	–	EA
St Peter (2010) [6]	Randomized controlled trial	< 18	Abscess	Well-circumscribed abscess on CT	Imaging	IV AB + drainage	LA at presentation	40	20	20	10.1 (4.2)	8.8 (4.2)	10 weeks	–	–
Surana (1995) [19]	Retrospective cohort	< 15	Mass	Mass not further defined	Palpable mass and/or imaging	IV AB	Appendectomy after diagnosis	198	189	9	–	–	4–6 weeks	–	NOT + IA
Tanaka (2016) [5]	Prospective cohort	< 18	Both	Appendix in the middle of well-circumscribed abscess or phlegmon on imaging	Imaging	IV AB + drainage (if necessary)	LA within 48 h after presentation	88	55	33	9.3 (3.0)	9.1 (3.3)	Mean 3.4 (± 1.7) years	Mean 3.2 (± 1.9) years	NOT

Data are expressed as n or mean (SD)

*AB* antibiotics, *EA* Early appendectomy, *IA* Interval appendectomy, *LA* Laparoscopic appendectomy, *NOT* initial non-operative treatment, *US* Ultrasound, *CT* Computed Tomography

### Quality of the studies

The interrater reliability for overall judgment of risk of bias was good (86% agreement). In only 14% of cases a third author was consulted. The interrater reliability for judging the subdomains of the ROBINS-I and Risk of Bias tool 2.0 was substantial (76% agreement). All studies were assessed as moderate to serious risk of bias on the primary outcome (overall complication rate) according to the ROBINS-I tool and some concerns were expressed according to the Risk of Bias tool 2.0 for the study by St Peter (Table 2) [5, 6, 12–23]. Bias due to confounding was serious in most cohort studies and moderate in only 4 of them [5, 13, 18, 21]. These four studies adjusted for baseline characteristics and all participants were preoperatively selected for inclusion.

Risk of bias in selection of participants was low in eight [5, 12–14, 18, 19, 21, 22], moderate in two [16, 20] and serious in two studies [17, 23].

Evidence and conclusions of the original studies were rated as level 2b in 3 studies [5, 6, 18] and level 4 in 11 studies [12, 13, 15–23].

**Table 2:** Risk of Bias on the primary outcome (overall complication rate) in included studies

Article	Risk of bias arising from the randomization process		Risk of bias due to deviations from the intended interventions	Missing outcome data	Risk of bias in measurement of the outcome	Risk of bias in selection of the reported result	Overall risk of bias	
St Peter (2010) [6]*	Low		Low	Low	Some concerns	Unclear	Some concerns	

Article	Bias due to confounding	Bias in selection of participants into the study	Bias in classification of interventions	Bias due to deviations from intended interventions	Bias due to missing data	Bias in measurement of outcomes	Bias in selection of the reported result	Overall Risk of Bias
Calvert (2014) [16]	Serious	Moderate	Moderate	Low	Low	Moderate	NI	Serious
Emil (2007) [17]	Serious	Serious	Serious	Low	Moderate	Moderate	NI	Serious
Erdogan (2004) [20]	Serious	Moderate	Moderate	Low	Low	Moderate	NI	Serious
Furuya (2015) [21]	Moderate	Low	Moderate	Low	Low	Moderate	Moderate	Moderate
Gahukamble (1993) [22]	Serious	Low	Moderate	Low	Serious	Moderate	NI	Serious
Gästrin (1969) [23]	Serious	Serious	Serious	Low	Moderate	Moderate	NI	Serious
Gillick (2001) [12]	Serious	Low	Moderate	Low	Low	Moderate	NI	Serious
Handa (1997) [13]	Moderate	Low	Moderate	Low	Low	Moderate	Moderate	Moderate
Puri (1981) [14]	Serious	Low	Serious	Low	Low	Moderate	NI	Serious
Roach (2007) [15]	Serious	Moderate	Moderate	Low	Low	Moderate	Moderate	Serious
Samuel (2002) [18]	Moderate	Low	Low	Low	Low	Moderate	Moderate	Moderate
Surana (1995) [19]	Serious	Low	Moderate	Low	Low	Moderate	NI	Serious
Tanaka (2016) [5]	Moderate	Low	Low	Low	Low	Moderate	NI	Moderate

^*^The Cochrane Collaboration’s Risk of Bias Tool for randomized controlled trials was applied for this study. Risk of bias for all other studies was assessed with the Risk of Bias in Non-randomized Studies of Interventions tool (ROBINS-1)

*NI* No Information

### Primary outcome: Overall complication rate

**Fig. 2:**
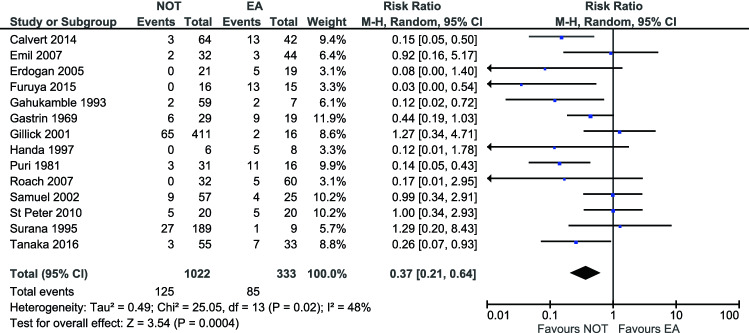
Overall complication rate

A total of 1355 patients were included in the analysis, of which 1055 were treated initially non-operatively and 333 underwent EA. In the NOT group 125 of 1022 patients (12.2%) experienced a complication. In the EA group a complication occurred in 85 of 333 patients (25.5%). Most reported complication in the NOT group was failure of NOT (80% of patients with a complication). For the EA group IAA was the most common complication (48% of patients with a complication). Due to the lack of available data it was not possible to stratify complications according to the Clavien–Dindo scale [8]. Meta-analysis showed that statistical heterogeneity between studies was substantial (*I*^2^ = 48%, *p* = 0.02). (Fig. 2.) The overall complication rate was significantly lower for initial NOT compared to EA (RR 0.37 [95%CI 0.21–0.64], *p* = 0.0004) [5, 6, 12–23]. Both the sensitivity analysis including only studies published after 2000 and the sensitivity analysis including only studies that further specified their definition of appendiceal abscess and/or mass (and thus not only mentioned the terms ‘appendiceal mass/phlegmon’ or ‘appendiceal abscess’), showed similar results [5, 6, 12, 13, 15–18, 20, 21]. When only studies with moderate risk of bias were included in the analysis, the effect was no longer statistically significant (RR 0.31 [95%CI 0.09–1.08], *p* = 0.07) (Table 3) [5, 6, 13, 18, 21].

**Table 3:** Results of meta-analyses of early appendectomy vs non-operative treatment on primary and secondary outcomes

Outcome	No. of studies	Total participants	Participants NOT	Participants EA	Heterogeneity *I*^*2*^, %	Risk Ratio (95% CI)	*p *value
*Primary outcome*							
Overall complications [5, 6, 12–23]	14	1355	1022	333	48	0.37 (0.21–0.64)	0.0004
*Subgroup mass/abscess*							
Overall complications (only mass) [12, 19, 20, 22]	4	731	680	51	56	0.44 (0.11–1.80)	0.25
Overall complications (only abscess) [6, 13, 21, 23]	4	133	71	62	63	0.33 (0.09–1.17)	0.09
Overall complications (combination of mass and abscess) [5, 14–18]	6	491	271	220	47	0.31 (0.14–0.69)	0.004
*Subgroup type of complication*							
IAA [5, 6, 12, 14–17, 19–23]	12	1253	953	300	16	0.32 (0.16–0.63)	0.001
Wound infection [12–14, 16–22]	10	1081	880	201	0	0.13 (0.06–0.31)	< 0.00001
Ileus [5, 6, 12–14, 16, 19, 21, 23]	9	993	815	178	0	0.20 (0.07–0.54)	0.001
*Sensitivity analyses*							
Overall complications (RoB)^a^ [5, 6, 13, 18, 21]	5	255	154	101	62	0.39 (0.13–1.17)	0.09
Overall complications (further specified definition)^b^ [5, 6, 13, 15–17, 21]	7	447	225	222	48	0.29 (0.12–0.71)	0.007
Overall complications (studies published after 2000) [5, 6, 12, 15–18, 20, 21]	9	982	708	274	55	0.42 (0.19–0.90)	0.03
*Secondary outcomes*							
Readmission rate [5, 12, 13, 15, 17, 19, 20, 22]	8	1001	805	196	0	1.75 (0.79–3.89)	0.17
*Sensitivity analyses*							
Readmission rate (RoB)^a^ [5, 13]	2	102	61	41	21	1.89 (0.43–8.44)	0.40
Readmission rate (studies published after 2000) [5, 12, 15, 17, 20]	5	723	551	172	16	2.15 (0.72–6.39)	0.17

^a^Sensitivity analysis including only studies with a moderate risk of bias

^b^Sensitivity analysis including only studies that reported a clear definition of appendiceal mass and/or abscess

Data are expressed as n or mean, unless otherwise specified

*RoB* risk of bias, *IAA* intra-abdominal abscess

### Subgroup analyses of the primary outcome

**Fig. 3:**
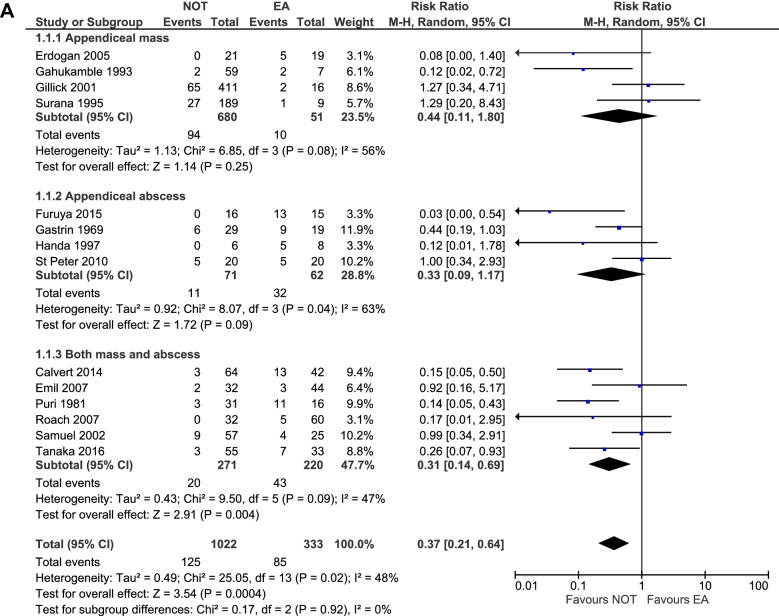

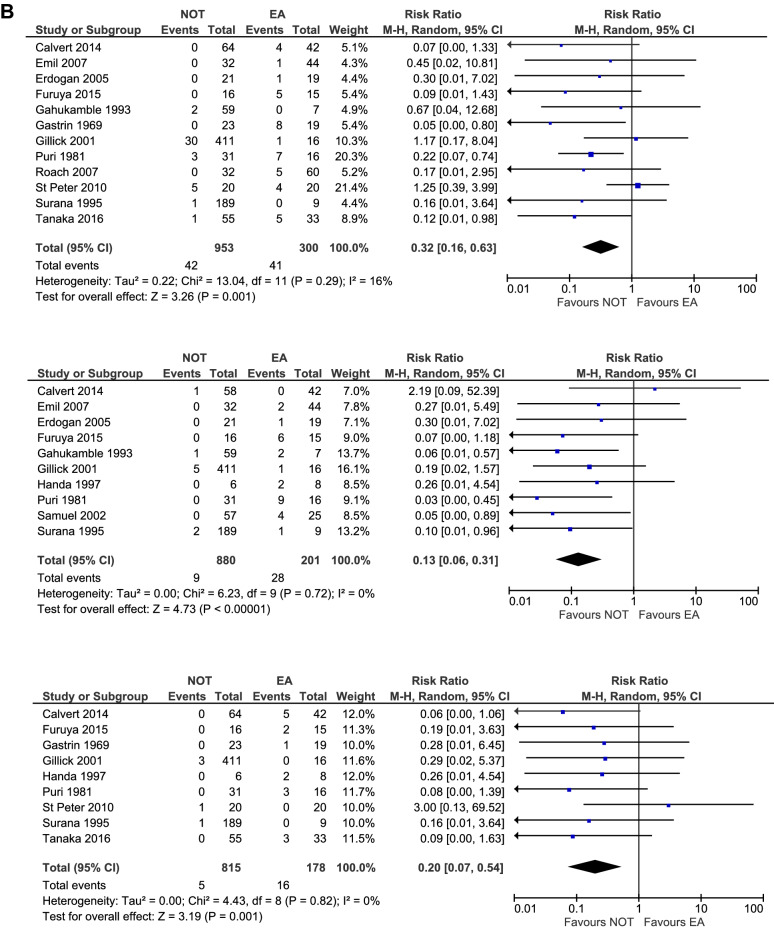
**a** Forest plot of subgroup analyses on overall complications. **b** Forest plot on subgroup analyses on type of complication. Upper forest plot: IAA, middle forest plot: Wound infection, lower forest plot: Ileus

Four studies reported specifically on appendiceal mass (total 731 patients, NOT *n* = 680, EA *n* = 51) [12, 19, 20, 22]. In this subgroup 94 out of 680 patients (14%) and ten out of 51 patients (20%) experienced a complication after NOT and EA, respectively. Four other studies focused on appendiceal abscess (total 133 patients, NOT n = 71, EA n = 62) [6, 13, 21, 23]. Eleven out of 71 patients (15%) treated non-operatively and 32 out of 62 patients (52%) that underwent EA for appendiceal abscess experienced a complication. Therefore in both subgroups NOT was associated with a lower risk of overall complications, although not significant. The other six studies did not specify between appendiceal abscess and mass and thus included children with mass, abscess, and both mass and abscess (total 491 patients, NOT n = 271, EA n = 220). Subgroup analysis of these studies showed a significantly lower overall complication rate in the NOT group [5, 14–18]. (Table 3, Fig. 3) Complications were further divided into IAA, wound infection, and ileus, which were the most reported complications. (Table 3, Fig. 3) Risk ratio of developing one of these complications was significantly lower for the initial NOT group compared to the EA group (RR 0.32 [95%CI 0.16–0.63]; RR 0.13 [95%CI 0.06–0.31]; RR 0.20 [95%CI 0.07–0.54], respectively). (Table 3)

### Funnel plot

The funnel plot regarding our primary outcome, overall complication rate, shows the possibility of some publication bias. Especially small cohort studies that favor EA are missing in current literature. (Online Appendix 3)

### Grade Evidence table

The certainty of the evidence regarding the overall complication rate was considered to be very low according to the GRADE principles. (Table 4)

**Table 4:** Grade Evidence Table Question: Non-operative treatment compared to early appendectomy for appendiceal mass and/or abscess in children

Certainty assessment							№ of patients		Effect		Certainty	Importance
№ of studies	Study design	Risk of bias	Inconsistency	Indirectness	Imprecision	Other considerations	Non- operative treatment	Early appendectomy	Relative (95% CI)	Absolute (95% CI)		
Overall complication rate												
14	13 observational studies 1 pilot-RCT	very serious ^a^	serious ^b^	not serious	serious ^c^	publication bias strongly suspected ^d^	123/1022 (12.0%)	85/333 (25.5%)	**RR 0.34** (0.19 to 0.63)	**168** **fewer per 1.000** (from 207 fewer to 94 fewer)	⨁◯◯◯ VERY LOW	CRITICAL

Overall complication rate (sensitivity analysis at moderate risk of bias)												
5	4 observational studies 1 Pilot-RCT	not serious	serious ^b^	not serious	serious ^c^	publication bias strongly suspected ^d^	17/154 (11.0%)	34/101 (33.7%)	**RR 0.39** (0.13 to 1.17)	**205** **fewer per 1.000** (from 293 fewer to 57 more)	⨁◯◯◯ VERY LOW	CRITICAL

Readmission rate												
8	observational studies	very serious ^a^	not serious	not serious	serious ^c^	none	54/805 (6.7%)	10/196 (5.1%)	**RR 1.75** (0.79 to 3.89)	**38 more per 1.000** (from 11 fewer to 147 more)	⨁◯◯◯ VERY LOW	IMPORTANT

Readmission rate (sensitivity analysis at moderate risk of bias)												
2	observational studies	not serious	not serious	not serious	serious ^c^	none	14/61 (23.0%)	4/41 (9.8%)	**RR 1.89** (0.87 to 9.02)	**87 more per 1.000** (from 13 fewer to 782 more)	⨁◯◯◯ VERY LOW	IMPORTANT

^a^The majority of studies did not correct for any confounding variables. Furthermore, a serious risk of bias in selection of patients was found in most studies, because a part of the patients did not have a preoperative suspicion of appendiceal mass/abscess and thus were included in the study perioperatively

^b^Substantial heterogeneity was found between studies

^c^The confidence interval around the pooled effect estimate is very wide and do not overlap

^d^The funnel plot shows that there are no small cohort studies in favor of early appendectomy, whereas four small cohort studies favored initial non-operative treatment

### Secondary outcomes

#### Length of hospital stay

Only three studies accurately reported initial length of stay of a total of 210 patients (NOT n = 103, EA n = 67) [5, 18, 20]. The mean initial length of stay varied between 4.8 and 13.0 days in these populations. The difference in initial length of stay varied between 0.1 [20] and 5.9 days [18] in favor of the EA group. Only the study of Samuel reported a significantly longer initial length of stay for the non-operative group.

Total length of hospital stay was reported in nine papers and varied between 6.7 [6] and 28.6 days [21] for the NOT group and 4.8 [18] and 26.2 days [21] for the EA group [5, 6, 12, 13, 15–18, 21]. Mean difference varied between 0.20 [6] and 8.40 days [18] in favor of the EA group. After pooling of the results, statistical heterogeneity was 99% and therefore it was decided not to show the pooled effect estimate. The considerable heterogeneity could be caused by the very small standard deviations that were reported in two studies, of which one study found a large mean difference in length of stay in favor of the EA group [15, 18].

However, if only studies with moderate risk of bias were included, statistical heterogeneity remained higher than 70% (i.e., 79%) [5, 6, 13, 21].

#### Readmission rate

Readmission rate was described in eight studies and was not statistically different between intervention groups (n = 1001, RR 1.75 [95%CI 0.79–3.89], *p* = 0.17; *I*^*2*^ = 0; Table 3) [5, 12, 13, 15, 17, 19, 20, 22]. Both sensitivity analysis including only studies with moderate risk of bias, and sensitivity analysis including only studies published after 2000, did not alter this outcome (Table 3) [5, 12, 13, 15, 17, 20].

#### Recurrence rate

Interval appendectomy was part of the NOT strategy in all but one study [6, 12–23]. In this patient preference study, only 16 of 55 (29%) patients who underwent NOT, desired interval appendectomy [5]. Recurrence rate prior to interval appendectomy ranged from 0% [15] to 12.5% [5, 12–14, 17, 19–23]. Recurrence rate in patients treated with an initial NOT strategy without interval appendectomy was 34% within a mean ± SD follow-up period of 3.4 ± 1.7 years [5].

#### Imaging studies and usage of pain medication

Number and type of imaging studies and number of doses of pain medication were compared in only one study [6]. A lower number of CT-scans was performed in children that underwent EA versus NOT (mean ± SD 1.5 ± 0.7 versus 2.1 ± 1.1; *p* = 0.04). Number of doses of pain medication was not significantly different between groups (mean ± SD 9.7 ± 4.0 (EA group) versus 7.1 ± 15.8 (NOT group)).

#### Histopathological examination

Eight papers described the results of histopathological examination [5, 12, 13, 17–20, 22]. Unexpected findings were found in two studies [12, 18]. Both found carcinoid tumors in 2 out of 331 patients (0.6%) and 1 out of 48 (2.1%) patients, respectively.

#### Number of interventions

Interventions described in the included studies were EA, interval appendectomy, drainage procedures, placement of peripherally inserted central catheters (PICC-lines), and reoperations (e.g., due to adhesive small bowel obstruction) [5, 6, 12–23]. The number of interventions ranged between 1 and 2 per patient in both treatment groups.

#### Quality of life

None of the studies reported on Quality of Life.

## Discussion

In daily practice, there is still no consensus regarding the optimal treatment strategy for children presenting with an appendiceal abscess or mass due to complex appendicitis. Studies regarding this topic are scarce, of low quality and the heterogeneity between studies is substantial. Therefore results of these studies and our review should be interpreted with caution. But, based on the available low-quality data, it seems that initial NOT may reduce the overall complication rate compared to EA in the overall group (thus appendiceal abscess and mass), without significantly increasing neither the total length of hospital stay nor the readmission rate, but the evidence is very uncertain. Nonetheless, the scarce and low-quality evidence emphasizes the importance of well-designed high quality studies and, for in the meantime, shared decision making.

To our knowledge, this review was the first to focus solely on the treatment of the specific subgroup of pediatric patients with appendiceal mass and abscess. Three slightly comparable meta-analyses have been published on this topic. However, one of those focused on both the adult and pediatric population and performed a subgroup analysis for children with appendiceal mass and/or abscess [3]. The other two more recent meta-analyses focused on the treatment of complex appendicitis in the pediatric population but focused on complex appendicitis in general and therefore included both patients with mass and/or abscess but also those without (only free perforation)) [24, 25].

All three meta-analysis have comparable results, which are in line with our review. The first mentioned meta-analysis was published in 2010 and included seven studies, of which four that were also included in our meta-analysis. They found a lower overall complication rate for initial NOT as well (OR 0.21 [95%CI 0.11–0.38]) [3]. The two more recent studies performed a subgroup/sensitivity analysis for studies reporting on children with a mixture of appendiceal mass and/or abscess, including eight (of which two were included in our meta-analysis) and four studies (all included in our meta-analysis), respectively, and found a lower overall complication rate for initial NOT as well (OR 0.27 [95%CI 0.08–0.85] [24] and RR 0.06 [95%CI 0.02–0.23] [25]). Our review support these findings and contributes due to the fact that our meta-analysis displays a more accurate estimation of the overall effect size compared to the others. Our predefined inclusion criteria selected a less heterogeneous group of solely children with appendiceal mass and/or abscess and we included a total number of 14 studies, whereas previous meta-analyses only included four [25], seven [3], and eight studies [24] in a subgroup analysis of a mixture of children with appendiceal mass, abscess, and both.

Furthermore our review has also integrated a subgroup analysis for the patients with an appendiceal mass and abscess individually, which has not been done in previous studies. Interestingly, in our subgroup analyses the only group with a significantly different overall complication rate between initial NOT and EA was the group with a mixture of appendiceal mass, abscess and both. Although this may have been caused by a type two error, in our review no significant difference was found in the subgroups of children with only an appendiceal mass or appendiceal abscess.

Focusing on the secondary outcomes, we are the first to describe the number of interventions, imaging studies, and doses of pain medication. Other secondary outcomes such as readmission rate were previously described and found to be higher in the EA group, which could not be confirmed by our review. Additionally, our review found a longer total length of hospital stay after NOT, although results could not be pooled due to significant heterogeneity. This longer length of stay after NOT could be explained by the second admission that was scheduled for interval appendectomy in almost all included studies, as initial length of stay did not differ between treatment groups. This systematic review and meta-analysis could only include one study that did not routinely perform an interval appendectomy. This study found a recurrence rate of 34% in a group of 38 patients during a mean follow-up period of 3.4 ± 1.7 years [5]. However, recent studies, including a large randomized controlled trial, have shown that a wait-and-see approach is justified after non-operative treatment for appendiceal mass, as recurrence rates are low and unexpected findings (such as malignancies) are rarely found after interval appendectomy in the pediatric population [26–30]. This wait-and-see approach after NOT would possibly reduce the potential benefit of a shorter total length of hospital stay after EA.

Contrary to the pediatric population, initial NOT is the standard of care for adult patients presenting with appendiceal mass and or abscess. Systematic reviews and meta-analyses have shown that EA results in a significantly higher overall complication rate, and more specifically a greater incidence of ileus/bowel obstruction, IAA, and wound infection [3, 31]. In the pediatric population, opponents of the initial NOT strategy hypothesize that the omentum is relatively smaller and underdeveloped in young children. Therefore a contained appendiceal mass is rarely seen perioperatively, which should lead to a significantly lower postoperative complication rate in these children [32, 33]. Thus it can be hypothesized that EA might be preferable in young children, and on the other hand older children and adolescents might benefit from NOT. Although available evidence is limited, this hypothesis could not be validated by our review as a higher overall complication rate after EA was found for young children as well [14, 22].

Furthermore, it can be expected that differences in operation techniques (i.e., open versus laparoscopic appendectomy) can be of influence on the complication rate. Previous studies found a significantly lower complication rate after laparoscopic appendectomy (15.30%) compared to open appendectomy (29.33%) for complex appendicitis [34]. However, due to the limited data available, the EA group could not be divided into laparoscopic and open appendectomy in our systematic review and meta-analysis.

The most important and major concern of not only this review but all studies reporting on the treatment of appendiceal mass and abscess is the lack of consensus regarding the definition of both appendiceal mass and abscess. In addition the terms ‘mass’ and ‘phlegmon’ are frequently used as substitutes in the current literature. The lack of an uniform definition leads to considerable heterogeneity between studies [35, 36]. Therefore interpretation of results and especially comparison of different study populations is difficult. In our review we attempted to solve this problem by using predefined criteria for appendiceal mass and abscess in our selection process. However, most studies did not provide a detailed definition and only reported the terms appendiceal ‘mass’, ‘phlegmon’, and ‘abscess’. As a result we had to restrict our predefined criteria and included only studies that at least mentioned those terms to describe their study population. In our opinion, it is of utmost importance that consensus is reached regarding uniform definitions for appendiceal mass and abscess (e.g., through a Delphi study which we are currently planning) and that future studies make use of them. These uniform definitions can help to include comparable study populations in future studies, which are needed to draw proper conclusions regarding the optimal treatment strategy for children with appendiceal mass and abscess.

Apart from the mentioned lack of consensus regarding the definition of appendiceal mass, the results of this review are hampered by the heterogeneity between the included studies. Differences in methodology, in- and exclusion criteria, age, duration and type of antibiotics could all influence the outcome of the study. Several studies included both children with appendiceal mass and abscess, whereas others specified to one of both conditions. It was decided to pool data of both subgroups, because of the limited number of studies that reported specifically on one of the subgroups. Because of this limitation, subgroup analyses were performed for studies reporting on appendiceal mass, appendiceal abscess, and a combination of both.

In addition, differences in the diagnosis of appendiceal mass and abscess were found between studies. Whereas some studies only included patients that had ultrasound or CT-proven appendiceal mass and abscess, others included patients with a palpable mass or a mass that was found perioperatively. Moreover the majority of studies did not report the clinical status of patients at presentation, the size of the mass and abscess, and demographics and did not control for these confounders in their analysis. Due to their retrospective design and the aforementioned concerns, most studies were prone to significant selection bias.

Furthermore the majority of included studies were small retrospective cohort studies. Only two prospective cohort studies, and one pilot randomized controlled trial could be included in this review. All studies were assessed as having moderate to serious risk of bias. This illustrates the necessity of high quality prospective studies regarding this topic.

In conclusion, high quality evidence regarding the optimal treatment strategy for children presenting with appendiceal mass or abscess is missing and substantial heterogeneity exists between studies. Initial NOT of children with an appendiceal mass or abscess may reduce the overall complication rate compared to EA, but the evidence is very uncertain. The results of this review illustrate the necessity of a uniform definition of appendiceal mass and abscess, and subsequent large prospective studies are needed to determine the optimal treatment strategy for children presenting with an appendiceal mass or abscess.

## Electronic supplementary material

Below is the link to the electronic supplementary material.

Supplementary file1 (DOCX 13 kb)

Supplementary file2 (DOCX 102 kb)

Supplementary file3 (DOCX 15 kb)

Supplementary file4 (DOCX 12 kb)
