# Supplementary material for: Management of appendiceal mass and abscess in children; early appendectomy or initial non-operative treatment? A systematic review and meta-analysis
Source: Surg Endosc. 2020 Jul 24;34(12):5234–49. doi: 10.1007/s00464-020-07822-y (PMC7644542; doi:10.1007/s00464-020-07822-y)
Supplement: Supplementary file 1 — Supplementary file1 (DOCX 13 kb) [file 464_2020_7822_MOESM1_ESM.docx]

Appendix 1. Search strategy

Pubmed search (November 7^th^ 2019, 4560 results):

(("Appendix"[Mesh] OR "Appendicitis"[Mesh] OR "Appendectomy"[Mesh] OR appendix[tiab]

OR appendic*[tiab] OR appendec*[tiab]) AND ("Abscess"[Mesh] OR abscess*[tiab] OR

abcess*[tiab] OR infiltrat*[tiab] OR mass[tiab] OR complicat*[tiab] OR perforat*[tiab] OR

gangrenous[tiab]) AND (child*[tw] OR schoolchild*[tw] OR infan*[tw] OR adolescen*[tw] OR

pediatri*[tw] OR paediatr*[tw] OR neonat*[tw] OR boy[tw] OR boys[tw] OR boyhood[tw] OR

girl[tw] OR girls[tw] OR girlhood[tw] OR youth[tw] OR youths[tw] OR baby[tw] OR babies[tw]

OR toddler*[tw] OR teen[tw] OR teens[tw] OR teenager*[tw] OR newborn*[tw] OR

postneonat*[tw] OR postnat*[tw] OR perinat*[tw] OR puberty[tw] OR preschool*[tw] OR

suckling*[tw] OR picu[tw] OR nicu[tw])) NOT ("Case Reports"[Publication Type] OR

"addresses"[Publication Type] OR "biography"[Publication Type] OR "comment"[Publication

Type] OR "directory"[Publication Type] OR "editorial"[Publication Type] OR

"festschrift"[Publication Type] OR "interview"[Publication Type] OR "lectures"[Publication

Type] OR "legal cases"[Publication Type] OR "legislation"[Publication Type] OR

"letter"[Publication Type] OR "news"[Publication Type] OR "newspaper article"[Publication

Type] OR "patient education handout"[Publication Type] OR "popular works"[Publication

Type] OR "congresses"[Publication Type])

Embase.com search (November 7^th^ 2019, 4882 results)

(('appendix'/exp OR 'appendicitis'/exp OR 'appendectomy'/exp OR appendix:ab,ti OR appendic*:ab,ti OR appendec*:ab,ti) AND ('abscess'/exp OR abscess*:ab,ti OR abcess*:ab,ti OR infiltrat*:ab,ti OR mass:ab,ti OR complicat*:ab,ti OR perforat*:ab,ti OR gangrenous:ab,ti)

AND ('child'/exp OR 'adolescent'/exp OR child*:ab,ti OR schoolchild*:ab,ti OR infan*:ab,ti OR

adolescen*:ab,ti OR pediatri*:ab,ti OR paediatr*:ab,ti OR neonat*:ab,ti OR boy:ab,ti OR

boys:ab,ti OR boyhood:ab,ti OR girl:ab,ti OR girls:ab,ti OR girlhood:ab,ti OR youth:ab,ti OR

youths:ab,ti OR baby:ab,ti OR babies:ab,ti OR toddler*:ab,ti OR teen:ab,ti OR teens:ab,ti OR

teenager*:ab,ti OR newborn*:ab,ti OR postneonat*:ab,ti OR postnat*:ab,ti OR perinat*:ab,ti

OR puberty:ab,ti OR preschool*:ab,ti OR suckling*:ab,ti OR picu:ab,ti OR nicu:ab,ti)) NOT

'case report'/de
