# Supplementary figures and images for: Management of appendiceal mass and abscess in children; early appendectomy or initial non-operative treatment? A systematic review and meta-analysis
Source: Surg Endosc. 2020 Jul 24;34(12):5234–49. doi: 10.1007/s00464-020-07822-y (PMC7644542; doi:10.1007/s00464-020-07822-y)

Appendix 2. Forest plots of secondary outcomes


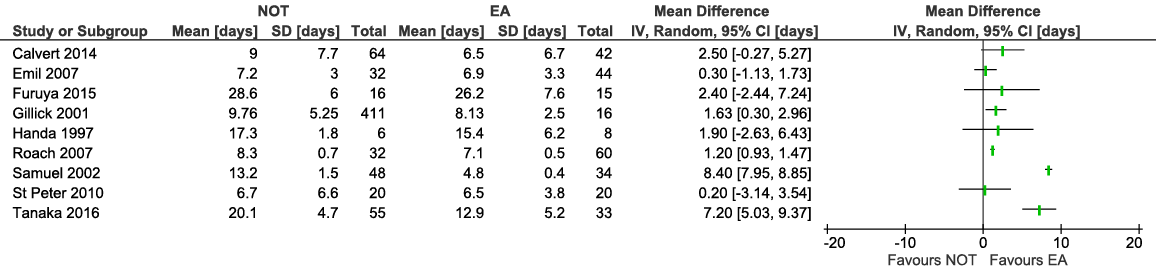


Total length of stay


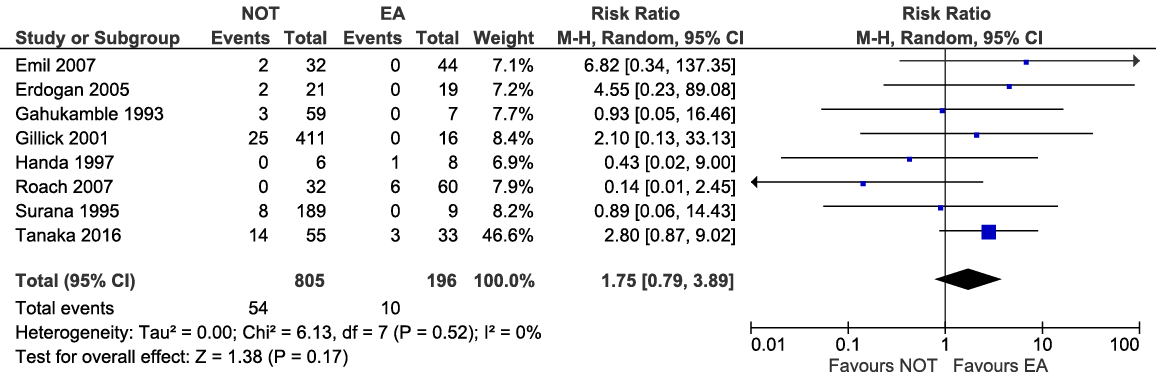


Readmission rate

Supplement: Supplementary file 2 — Supplementary file2 (DOCX 102 kb) [file 464_2020_7822_MOESM2_ESM.docx]

Appendix 3. Funnel plot overall complication rate


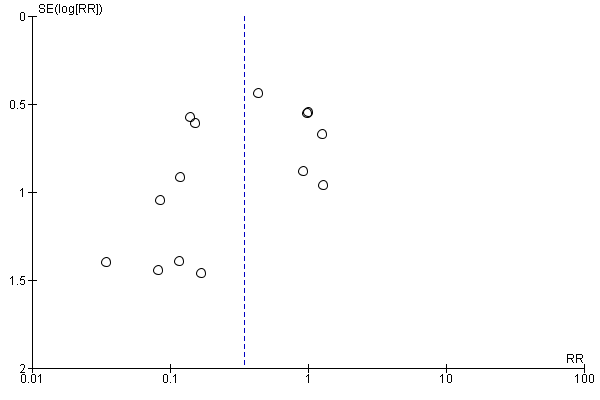

Supplement: Supplementary file 3 — Supplementary file3 (DOCX 15 kb) [file 464_2020_7822_MOESM3_ESM.docx]
