# Supplementary material for: Management of appendiceal mass and abscess in children; early appendectomy or initial non-operative treatment? A systematic review and meta-analysis
Source: Surg Endosc. 2020 Jul 24;34(12):5234–49. doi: 10.1007/s00464-020-07822-y (PMC7644542; doi:10.1007/s00464-020-07822-y)
Supplement: Supplementary file 4 — Supplementary file4 (DOCX 12 kb) [file 464_2020_7822_MOESM4_ESM.docx]

Appendix 4. Deviations from the registered protocol

During the review process we experienced that available literature on our research question was scarce. Therefore it was decided to include articles written in English, German, French, and Dutch, whereas our protocol stated that we would only include articles written in English.

Before the review process we decided to include all studies that evaluated early appendectomy as treatment for appendiceal mass or abscess with or without comparison to a control group of children that were initially treated non-operatively. As available literature was scarce and the majority of studies were retrospective cohort studies, selection bias was considered to be serious. To minimize the potential selection bias and improve comparability of studies, it was decided to only include studies that compared non-operative treatment with early appendectomy.

In our registered protocol we described that risk of bias in interventional cohort studies would be assessed with the Newcastle-Ottawa scale. However, as described in the protocol, the Cochrane collaboration’s tool for assessing risk of bias was used for to evaluate risk of bias in randomised controlled trials included in this systematic review and meta-analysis. The ROBINS-I tool assesses risk of bias on domains that are comparable to the Cochrane’s Risk of Bias tool that is used for randomised controlled trials. Therefore it was decided to use the ROBINS-I tool instead of the Newcastle Ottawa Scale to assess risk of bias in all cohort studies that were included in this systematic review and meta-analysis.

Additional subgroup analyses were performed on studies reporting on appendiceal abscess, appendiceal mass, and both. In our protocol we prespecified that we would not perform any subgroup analyses. However during the review process we realised that outcomes for these subgroups could differ substantially. Additionally, we decided to divide our primary outcome, overall complication rate, into the most frequently reported complications (intra-abdominal abscess, wound infection, and ileus) and perform subgroup analyses accordingly.

Sensitivity analyses on studies at moderate risk of bias were performed for all pooled outcome measures.
